# Supplementary material for: Enhancing bullfrog farming sustainability: circular water management through effluent treatment
Source: PeerJ. 2025 May 6;13:e19390. doi: 10.7717/peerj.19390 (PMC12063605; doi:10.7717/peerj.19390)
Supplement: Supplemental Information 1 [file peerj-13-19390-s001.zip › Supplementary files 1/Table 3.docx]

**Table 3** Tailwater treatment results (Raw monitoring data)

| Parameter | Tailwater1 | Tailwater2 | Tailwater3 | Effluent1 | Effluent2 | Effluent3 |
| --- | --- | --- | --- | --- | --- | --- |
| pH | 6.78 | 6.84 | 7.06 | 7.05 | 7.15 | 7.37 |
| Suspended solids (mg·L^−1^) | 1395.7 | 1425.2 | 1445.3 | 71.7 | 68.8 | 69.5 |
| Dissolved oxygen (mg·L^−1^) | 1.05 | 1.18 | 1.39 | 3.33 | 3.41 | 3.75 |
| COD_Cr_ (mg·L^−1^) | 121.7 | 115.8 | 122.5 | 32.6 | 35.6 | 33.8 |
| BOD (mg·L^−1^) | 90.5 | 95.7 | 113.8 | 9.7 | 10.1 | 10.2 |
| Ammoniacal nitrogen (mg·L^−1^) | 23.5 | 27.1 | 27.7 | 4.1 | 4.3 | 4.8 |
| Nitrite nitrogen (mg·L^−1^) | 0.31 | 0.35 | 0.36 | 0.021 | 0.028 | 0.032 |
| Total nitrogen (mg·L^−1^) | 25.5 | 29.1 | 29.4 | 16 | 16.5 | 18.5 |
| Reactive phosphorus (mg·L^−1^) | 13.8 | 16 | 15.2 | 1.8 | 2.3 | 1.9 |
| Total phosphorus (mg·L^−1^) | 30.2 | 35 | 36.8 | 7.3 | 9 | 7.7 |
| Color intensity (degree) | 982 | 1008 | 1010 | 1 | 3 | 3 |
| Odor intensity (level) | 3 | 4.5 | 4.5 | 1 | 0.5 | 1.5 |
